# First person – Avery Hinks

**DOI:** 10.1242/bio.059508

**Published:** 2022-07-25

## Abstract

First Person is a series of interviews with the first authors of a selection of papers published in Biology Open, helping early-career researchers promote themselves alongside their papers. Avery Hinks is first author on ‘ Influence of weighted downhill running training on serial sarcomere number and work loop performance in the rat soleus’, published in BiO. Avery is an MSc student (soon to be PhD student) in the lab of Dr Geoffrey Power at the University of Canada, investigating how muscle structure influences muscle function, particularly regarding adaptations following an intervention.


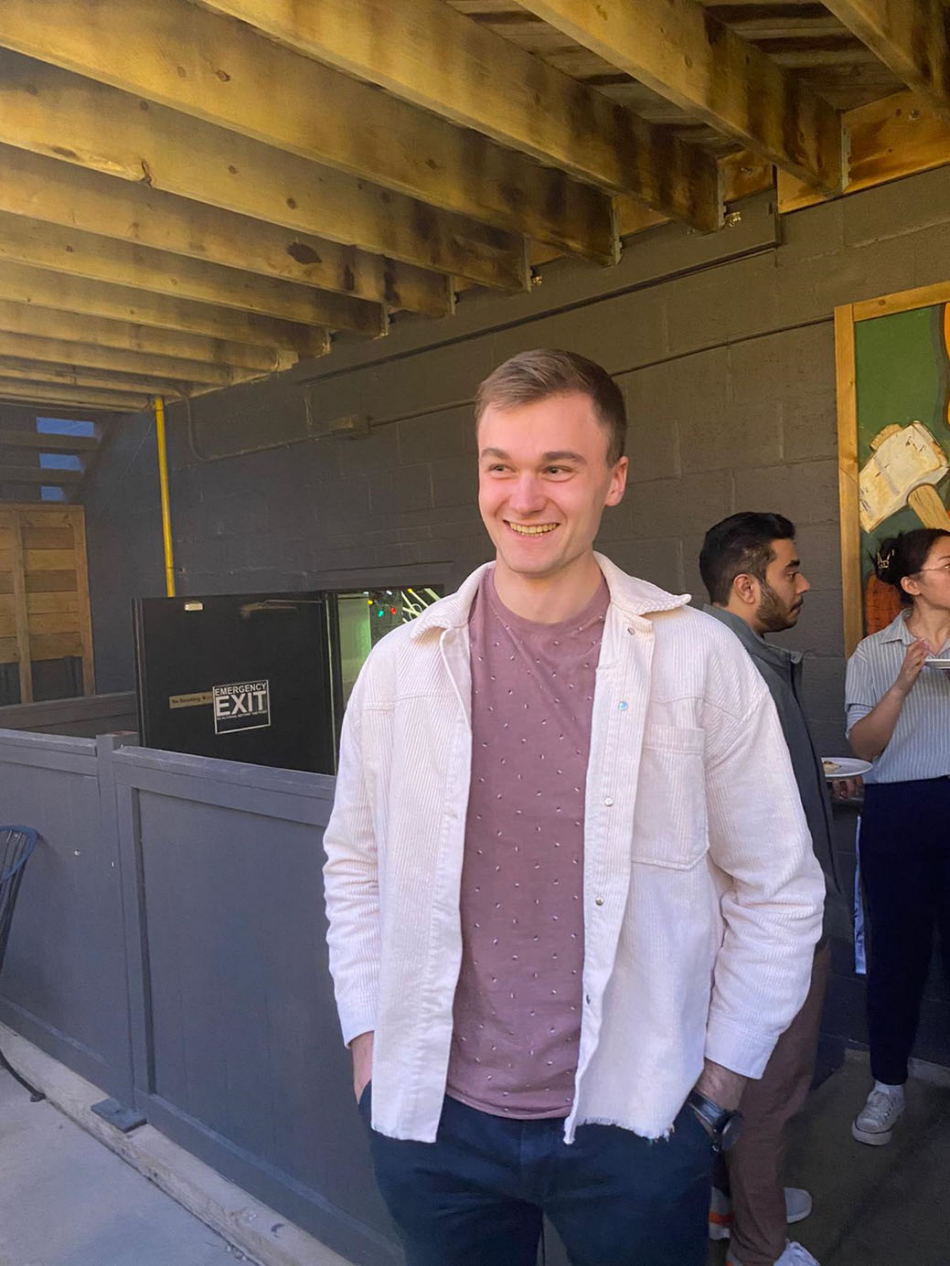


**Avery Hinks**

**Describe your scientific journey and your current research focus**

My research career began with a summer research assistantship in Dr Geoff Power's lab following the third year of my undergraduate degree in Human Kinetics at the University of Guelph. That summer Dr Power gave me the opportunity to run my own study on how training-induced adaptations in muscle architecture influence aspects of human muscle mechanical function, with the promise that I would get to write a first-author paper afterwards if I was interested. That project turned into my fourth-year research project, and the following summer (during covid) I also had the opportunity to publish old data from a previous graduate student's project. I had such a great time through all those experiences that I decided to stick around for a Master's degree. For my Master's, I switched to working with animals, but investigated similar ideas to my undergrad work in humans. Working with animals allowed me to learn about what goes on in muscle at much smaller scales. I intend to bring that knowledge into my PhD, where I am hoping to utilize both animal and human models to investigate how muscle structure influences muscle mechanical function.

**Who or what inspired you to become a scientist?**

I have a strange answer for this. I feel more like I ‘ended up’ where I am than I feel like I was inspired to pursue research as a career. Toward the end of my third year of undergrad, I was racking my brain trying to figure out what I wanted to do with my life, then I saw some postings for summer research assistantships and applied to 12 of them (in a variety of fields: student wellness, the campus library, psychology, physiology, and biomechanics). Dr Power offered me the position during my interview, so I took it! I'm still here because I've continued to be fulfilled and have had a genuinely great time. With all this said, there is likely a part of me that was inspired by films centered around scientists or professors that I watched growing up: Jurassic Park, Back to the Future, Raiders of the Lost Ark, The Fly, Ghostbusters, Cloudy With a Chance of Meatballs…who knows!

**How would you explain the main finding of your paper?**

Downhill running training can cause muscle to increase in length due to its emphasis on lengthening contractions. In my study, I wanted to see if weighted downhill running training would induce greater muscle lengthening adaptations than a previous study of body weight downhill running training in rats and assess the influence on muscle performance in dynamic movements. As predicted, I observed a greater incidence of muscle lengthening adaptations with weighted downhill running training compared to the previous body weight running study. There were also some improvements in dynamic muscle performance, however, they did not appear to be related to the muscle lengthening adaptations and seemed to be more related to adaptations in muscle strength.

**What are the potential implications of this finding for your field of research?**

Weighted downhill running training in rats may be used going forward as a stronger eccentric training stimulus compared to the more often used body weight downhill running training. As well, the connection between serial sarcomere number and dynamic muscle performance may not be as clear-cut as hypothesized.

**Figure:**
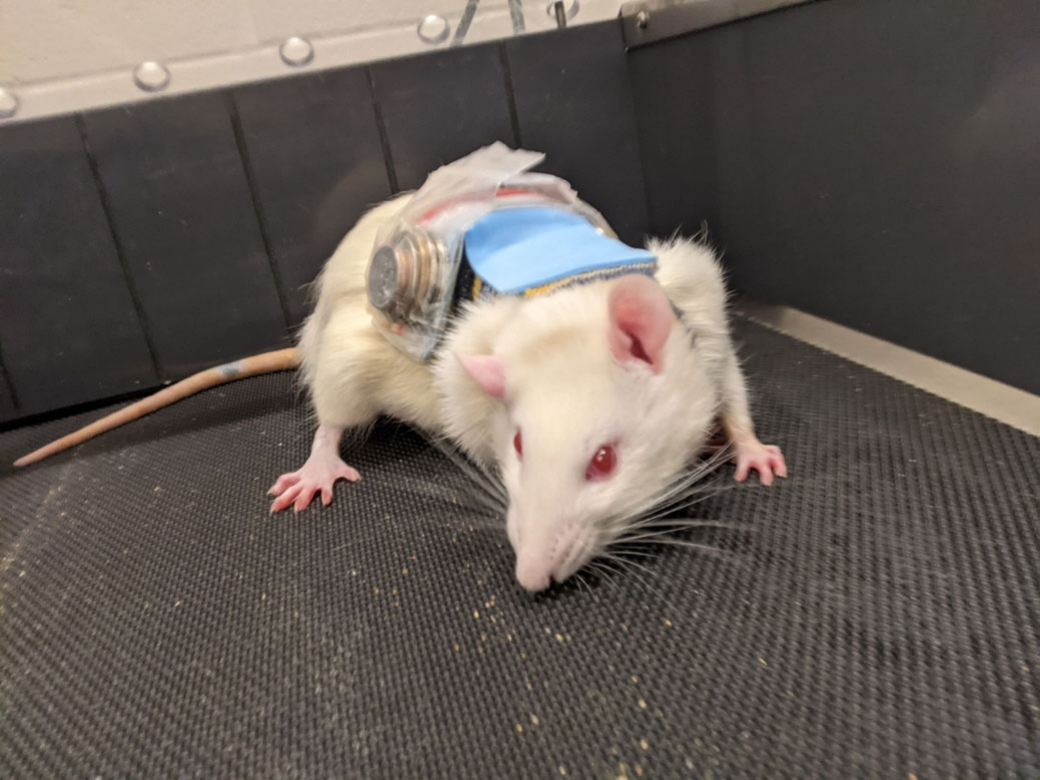
A rat wearing a weighted vest constructed from a child's size sock, foam paper, tiny Ziplock bags, and Canadian coins.

**Which part of this research project was the most rewarding?**

By far the most rewarding part of this project was making my rat weighted vests. As far as I know (and I looked everywhere to see if I could replicate someone else's methods), no previous study has done weighted vest running training in rats. It took extensive piloting and trial-and-error to nail down a vest design that was easy to put on the rats and did not impede their mobility during running. One day I found a YouTube video in which someone had trained their pet rats to play basketball wearing sweaters made from children's socks, and my creative mind ran wild. I am extremely proud of my final vest design, and to this day I still can't believe it actually worked.

“…I found a YouTube video in which someone had trained their pet rats to play basketball wearing sweaters made from children's socks, and my creative mind ran wild.”

**What do you enjoy most about being an early-career researcher?**

I enjoy that I get to wake up every day and investigate specific areas of research that only a small handful of people in the whole world are investigating. Perhaps that applies to being a scientist in general, but as of right now, so early in my career, that is what I am most thrilled by.

**What piece of advice would you give to the next generation of researchers?**

Get eight hours of sleep per night and have hobbies.

**What's next for you?**

I'm staring a PhD in Dr Power's lab in September!
